# Modulation of Toxin Stability by 4-Phenylbutyric Acid and Negatively Charged Phospholipids

**DOI:** 10.1371/journal.pone.0023692

**Published:** 2011-08-22

**Authors:** Supriyo Ray, Michael Taylor, Mansfield Burlingame, Suren A. Tatulian, Ken Teter

**Affiliations:** 1 Department of Physics, University of Central Florida, Orlando, Florida, United States of America; 2 Burnett School of Biomedical Sciences, College of Medicine, University of Central Florida, Orlando, Florida, United States of America; 3 Lake Brantley High School, Altamonte Springs, Florida, United States of America; Institute Pasteur, France

## Abstract

AB toxins such as ricin and cholera toxin (CT) consist of an enzymatic A domain and a receptor-binding B domain. After endocytosis of the surface-bound toxin, both ricin and CT are transported by vesicle carriers to the endoplasmic reticulum (ER). The A subunit then dissociates from its holotoxin, unfolds, and crosses the ER membrane to reach its cytosolic target. Since protein unfolding at physiological temperature and neutral pH allows the dissociated A chain to attain a translocation-competent state for export to the cytosol, the underlying regulatory mechanisms of toxin unfolding are of paramount biological interest. Here we report a biophysical analysis of the effects of anionic phospholipid membranes and two chemical chaperones, 4-phenylbutyric acid (PBA) and glycerol, on the thermal stabilities and the toxic potencies of ricin toxin A chain (RTA) and CT A1 chain (CTA1). Phospholipid vesicles that mimic the ER membrane dramatically decreased the thermal stability of RTA but not CTA1. PBA and glycerol both inhibited the thermal disordering of RTA, but only glycerol could reverse the destabilizing effect of anionic phospholipids. In contrast, PBA was able to increase the thermal stability of CTA1 in the presence of anionic phospholipids. PBA inhibits cellular intoxication by CT but not ricin, which is explained by its ability to stabilize CTA1 and its inability to reverse the destabilizing effect of membranes on RTA. Our data highlight the toxin-specific intracellular events underlying ER-to-cytosol translocation of the toxin A chain and identify a potential means to supplement the long-term stabilization of toxin vaccines.

## Introduction

Cholera toxin (CT), pertussis toxin (PT), Shiga toxin (ST), and the plant toxin ricin are AB-type protein toxins that contain a catalytic A subunit and a receptor-binding B subunit [1], [2]. These toxins move from the cell surface to the endoplasmic reticulum (ER) as intact holotoxins. Conditions in the ER promote the dissociation of the catalytic A subunit from the rest of the toxin [3]–[7]. Unfolding of the isolated toxin A chain subsequently activates the quality control mechanism of ER-associated degradation (ERAD) [2]. This system recognizes misfolded or misassembled proteins in the ER and exports them to the cytosol through one or more protein-conducting channels in the ER membrane [8]. Most exported ERAD substrates are degraded by the ubiquitin-26S proteasome system, but ER-translocating toxins avoid this fate because their lysine-poor A chains lack the target amino acid residue for ubiquitin conjugation [9]–[12]. Instead, the translocated A chain refolds in the cytosol and modifies its intracellular target to initiate the cellular effects of intoxication.

ER-translocating toxins were originally thought to masquerade as misfolded proteins in order to activate the ERAD translocation mechanism [10]. However, accumulating evidence suggests the toxin A chain actually assumes an unfolded conformation after dissociation from the holotoxin. The isolated A chains of both CT (CTA1) and PT (PT S1) are in disordered conformations at the physiological temperature of 37°C [13]–[15]. Ricin toxin A chain (RTA) is more stable than CTA1 or PT S1 [16]–[18], but its unfolding in the ER is promoted by an interaction with negatively charged phospholipids. This was originally demonstrated using unilamellar vesicles enriched with the anionic phospholipid 1-hexadecanoyl-2-(9Z-octadecenoyl)-*sn*-glycero-3-phospho-(1′-*rac*-glycerol) (POPG) and was later demonstrated with ER-derived microsomes [19], [20]. Membrane interaction appears to involve a hydrophobic stretch of amino acids near the C-terminus of RTA [20], [21]. The C-terminal region of the ST A1 subunit (STA1) also interacts with negatively charged vesicles and is actively involved with the ER-to-cytosol translocation event [22]–[25]. A potential destabilizing interaction between anionic phospholipids and CTA1 or PT S1 has not yet been examined.

AB toxins that enter the cytosol from acidified endosomes utilize a pH-dependent mechanism for A chain translocation to the cytosol [1]. In contrast, exposure to acidic pH is not required for productive intoxication with either CT or ricin [26]–[28]. Both travel as intact holotoxins from the cell surface to the endosomes, from the endosomes to the *trans*-Golgi network, and from the *trans*-Golgi network to the ER (Fig. S1) [29], [30]. A/B subunit dissociation occurs in the ER, which maintains a near-neutral pH similar to the cytosolic pH of 7.1–7.4 [31], [32]. The holotoxin-associated A chains are held in stable conformations [33], [34], but unfolding of the dissociated CTA1 subunit occurs at 37°C and pH 7.0–7.4 [14], [35]. Unfolding of the isolated RTA subunit likewise occurs at 37°C and pH 7.1 in the presence of anionic phospholipids which mimic the ER membrane [19]. Although acidic pH often denatures proteins, we have reported that a pH 6.0 buffer actually stabilizes the CTA1 subunit [36]. A pH 6.5 buffer also prevents the thermal unfolding of CTA1, while a pH 8.5 buffer destabilizes CTA1 (A.H. Pande and K. Teter, unpublished observations). Acidic pH has also been reported to stabilize RTA [16]. The mildly acidic conditions in the early endosomes and *trans*-Golgi network [31], [37] are therefore unlikely to begin the unfolding process before holotoxin transport to the ER. As such, negatively charged phospholipids and/or physiological temperature appear to be the main contributing factors for unfolding of the dissociated CTA1 and RTA subunits.

In addition to its role in ERAD-mediated translocation, the instability of RTA also impacts the process of vaccine development. Efforts to produce a vaccine against ricin, a potential bioterror agent [38], have been hampered by the negative impact of RTA instability on its expression and storage [17], [39]–[42]. Thus, recombinant variants of RTA with increased thermostability have been generated as potential vaccine candidates [17], [42]. Protein stabilizers have also been evaluated as potential additions to a RTA vaccine [18]. Recently, a mutant RTA-based vaccine (RiVax) in clinical evaluation [43], [44] has been shown to maintain immunogenicity for one year when lyophilized in the presence of 20% trehalose [45].

We have previously suggested that the inhibition of A chain unfolding represents a potential target for broad-spectrum anti-toxin therapeutics [35]. Stabilization of the dissociated A chain would prevent its recognition by the ERAD system, its ER-to-cytosol export, and, thus, its toxic effect in the cytosol. We demonstrated this principle with 4-phenylbutyric acid (PBA), a chemical chaperone and therapeutic agent approved by the Food and Drug Administration (FDA) for the treatment of urea cycle disorders [46]. PBA inhibited the thermal disordering of CTA1, the ER-to-cytosol translocation of CTA1, and CT activity against both cultured cells and ileal loops [47]. In this work, we examined whether PBA also blocks the thermal disordering of RTA. Two medicinal benefits could result from the potential stabilization of RTA by PBA: (i) an extended shelf-life for recombinant RTA vaccines; and (ii) an inhibition of the intoxication process via disruption of ERAD-mediated translocation to the cytosol.

The overall aim of this work was to assess the medicinal value of PBA as a protein stabilizer for RTA and as a ricin inhibitor. Biophysical studies demonstrated that PBA binds to RTA and increases the thermal stability of the protein without affecting its structure. PBA or similar small molecules could thus potentially be used to improve RTA vaccine production by preserving its long-term conformational stability. However, PBA did not inhibit ricin intoxication of cultured cells. Additional experiments demonstrated that the destabilizing effect of anionic phospholipids on RTA structure was dominant over the stabilizing effect of PBA. In contrast, negatively charged phospholipids did not destabilize CTA1 and did not prevent the thermal stabilization of CTA1 by PBA. These collective results provide a molecular explanation for why PBA protects cells from CT but not ricin: PBA will stabilize CTA1, but not RTA, in the presence of the negatively charged phospholipids of the ER membrane. Our data, which highlight the importance of toxin-phospholipid interactions for the translocation of RTA, demonstrate that distinct ERAD-related events are active in the export of different ER-translocating toxins.

## Materials and Methods

### Materials

Ricin holotoxin, ricin B chain, and RTA were purchased from Vector Laboratories, Inc. (Burlingame, CA). Culture supernatant from ST1- and ST2-producing *Escherichia coli* O157 strain RM1697 was kindly provided by Dr. Beatriz Quinones (US Department of Agriculture, Western Regional Research Center). CTA1-His_6_ was purified as previously described [36]. 1-palmitoyl-2-oleoyl-*sn*-glycero-3-phosphocholine (POPC) and POPG were purchased from Avanti Polar Lipids (Alabaster, Alabama). PBA was purchased from EMD Chemicals (Gibbstown, NJ); Na_2_HPO_4_, KH_2_PO_4_, and NaCl were purchased from Fisher Scientific (Pittsburgh, PA); chloroform and ethanolamine were purchased from Sigma Aldrich (St. Louis, MO); and NHS and EDC were purchased from Thermo Scientific (Rockford, IL). Rabbit anti-RTA and anti-ricin B chain antibodies were purchased from Abcam (Cambridge, MA).

### Preparation of large unilamellar vesicles (LUVs)

Lipid solutions of 10 mM POPC were made in chloroform, and lipid solutions of 10 mM POPG were made in chloroform:methanol (2∶1, v/v). After mixing POPC and POPG solutions in a 4∶1 molar ratio, the solvent was evaporated under a steady stream of nitrogen gas and then placed in a desiccator for 4 h. To prepare LUVs, the dried lipid mixture was suspended in 10 mM Na/K phosphate buffer (pH 7.2) containing 50 mM NaCl, vortexed thoroughly, and extruded at room temperature through 100 nm pore size polycarbonate membranes using a LiposoFast extruder (Avestin, Ottawa, Canada) with 30 passes.

### Surface plasmon resonance (SPR)

A Reichert (Depew, NY) SR7000 SPR Refractometer with a flow rate of 41 µl/min was used for SPR experiments. To create a sensor slide, we activated a self-assembled monolayer gold sensor slide (Reichert) by a 10 min perfusion with a buffer containing 0.4 M EDC and 0.1 M NHS. Phosphate-buffered saline (pH 7.4) containing 0.05% Tween 20 (PBST) was passed over the plate for 5 min to remove the activation buffer. An anti-RTA antibody at 1∶1,000 dilution in 20 mM sodium acetate (pH 5.5) was then perfused over the slide for 10 min. Following another 5 min PBST wash, the remaining active sites on the slide were deactivated with a 3 min perfusion of 1 M ethanolamine pH 8.5. A baseline measurement corresponding to the mass of the bound antibody was taken, and 20 µg/ml RTA in PBST was then passed over the antibody-coated plate for 5 min. The increased refractive index unit (RIU) resulting from antibody capture of RTA confirmed the presence of toxin on the plate. This elevated RIU reading was set as the new baseline value before perfusion of 100 µM PBA over the toxin-bound sensor slide. Reichert Labview software was used for data collection, and the BioLogic (Campbell, Australia) Scrubber 2 software was used for data analysis.

For experiments involving ricin holotoxin or ricin B chain, the aforementioned protocol was followed except the activated plate was initially exposed to a 1∶500 dilution of anti-ricin B chain antibody. Either 20 µg/ml of ricin holotoxin or 20 µg/ml of ricin B chain was then used to coat the plate. The baseline value resulting from toxin deposition on the sensor was lower for holotoxin-coated plates than RTA-coated plates, indicating that the holotoxin-coated sensor had less toxin appended to it than the RTA-coated sensor. This could be due to a difference in the quality of the anti-RTA vs. anti-RTB antibodies used to append RTA and ricin holotoxin, respectively, to their sensors. The different toxin quantities on the sensor slides affected the arbitrary RIU signal but would not affect the affinity of PBA-toxin interactions [48].

### Circular dichroism (CD) and fluorescence spectroscopy

For measurements of RTA secondary structure, far-UV CD experiments with a Jasco 810 spectrofluoropolarimeter were conducted on 66 µg/ml of RTA (2 µM) in 10 µl Na/K phosphate buffer (pH 7.2) with 1 mM dithiothreitol. The toxin was placed in a 0.1 mm optical path-length quartz cuvette (Hellma USA, Plainview, NY) and heated from 20°C to 60°C in 2°C increments using a Neslab RTE 7 thermostat (Thermo Fisher Scientific, Waltham, MA). The sample was equilibrated for 4 min at each temperature before measurement. Where indicated, the sample was placed in a buffer containing 10% glycerol, 100 µM PBA, and/or 600 µM LUVs containing 80% POPC and 20% POPG. Additions were made at 20°C before the measurements. In experiments involving a combination of PBA and POPC:POPG LUVs, RTA was incubated with PBA for 30 min at 20°C before the addition of LUVs and commencement of measurements. Likewise, RTA was pre-incubated with 10% glycerol for 30 min at 20°C before the addition of LUVs and commencement of measurements.

The temperature-dependence of CTA1 structure was studied by CD and fluorescence techniques as previously described [14], [35], [36], [47]. Sample concentration was 72 µg of CTA1-His_6_ in 220 µl of 10 mM sodium borate buffer (pH 7.2), or 15 µM, in a 4 mm × 4 mm rectangular quartz cuvette. CTA1 was heated from 20°C to 60°C in 1°C or 2°C increments using a Jasco PFD-425S Peltier temperature controller. At each temperature, the sample was incubated for 4 min before measurement. Toxin samples incubated with PBA, LUVs, or both PBA and LUVs were prepared as described above for RTA, including the 30 min pre-incubation with PBA before addition of POPC:POPG LUVs. Thermal unfolding profiles for both CTA1 and RTA were calculated as previously described [14], [35], [36], [47].

### Toxicity assay

As previously described [49], Vero cells expressing a destabilized variant of the enhanced green fluorescent protein (Vero-d2EGFP) were seeded in 96-well microplates and exposed to varying concentrations of ricin or culture supernatant from ST1- and ST2-producing *E. coli* O157 strain RM1697 [50] for 16 hr at 37°C in a 5% CO_2_ humidified incubator. EGFP fluorescence was then measured on a Synergy HT Multi-Detection Microplate Reader (BioTek, Winooski, VT) with the 485/20 nm excitation filter and the 528/20 nm emission filter. Results from toxin-treated cells were expressed as percentages of the values obtained from control cells incubated without toxin.

## Results

**Figure 1:**
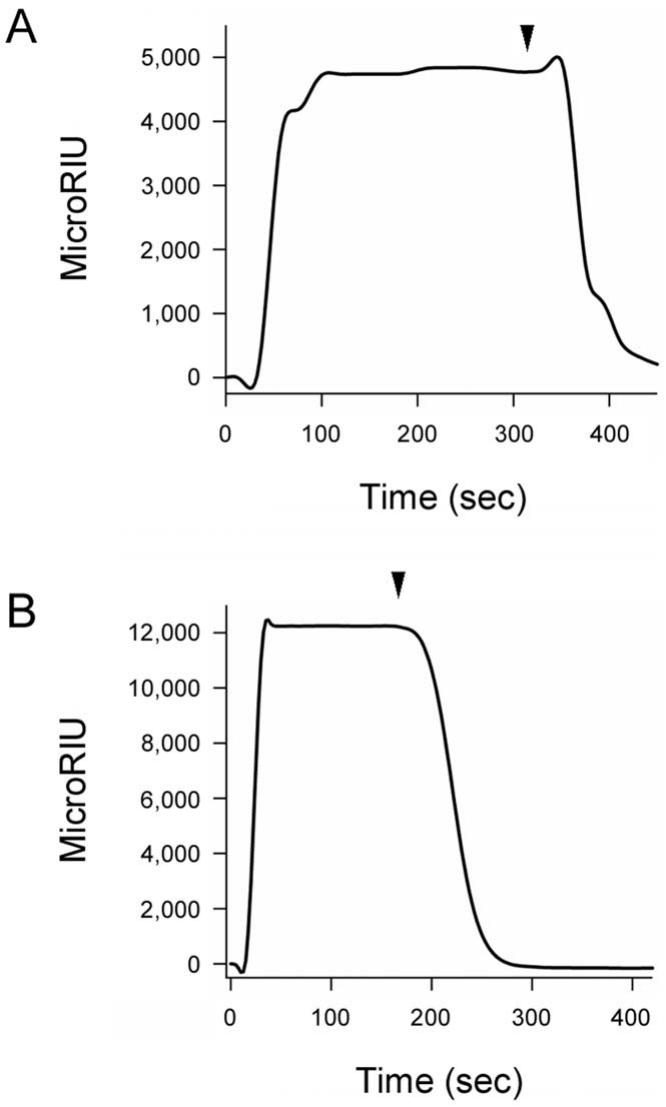
PBA binds to ricin. PBA was perfused at a 100 µM concentration over SPR sensor slides coated with ricin holotoxin (A) or RTA (B). For each slide, the baseline signal from the bound toxin was set at 0 microRIU before PBA perfusion. Arrowheads indicate the time at which PBA was removed from the perfusion buffer.

We have recently reported that PBA binds directly to CTA1 and prevents its thermal unfolding [47]. To determine whether PBA could also bind to RTA, we used the technique of SPR. PBA was perfused over SPR sensor slides coated with ricin holotoxin or RTA (Fig. 1). A positive signal was detected with both the holotoxin (Fig. 1A) and the isolated A chain (Fig. 1B). A stronger signal in the case of RTA compared to the holotoxin likely indicates a higher surface density of the protein in the former case. Preliminary calculations of the association rate constants (*k*_a_) for these experiments provided further support for this interpretation, as PBA binding to both RTA and ricin holotoxin was characterized by similar values of *k*_a_. The rapid return of the signal to baseline value upon removal of PBA from the perfusion buffer indicated that PBA binding to either RTA or the holotoxin is easily reversible. No signal was obtained when ricin B chain was exposed to PBA (not shown), which strongly suggested that PBA binding to the ricin holotoxin resulted from an interaction with the holotoxin-associated A chain. Far-UV CD confirmed that our ricin B chain was in the folded conformation expected from previous reports (not shown) [51].

**Figure 2:**
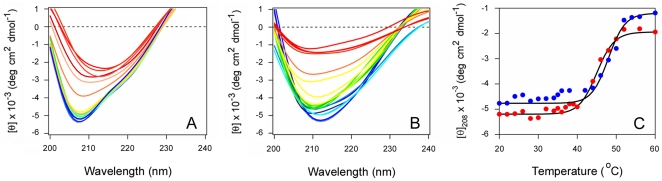
PBA inhibits the thermal unfolding of RTA. (A–B): Far-UV CD measurements of RTA secondary structure were taken in the absence (A) or presence (B) of 100 µM PBA. Data were recorded with 2 µM RTA in pH 7.2 buffer. The change in color from blue to red corresponds to a change in temperature from 20°C to 60°C. (C): The mean residue molar ellipticities at 208 nm ([θ]_208_) in the absence (red) or presence (blue) of PBA were plotted as a function of temperature.

Far-UV CD was used to determine the impact of PBA binding on the structural stability of RTA (Fig. 2). Measurements of protein secondary structure were taken at defined temperatures as the A chain was heated from 20°C to 60°C in either the absence (Fig. 2A) or presence (Fig. 2B) of 100 µM PBA. For both conditions, RTA exhibited a strong minimum between 207–212 nm and a weaker shoulder around 220–222 nm. These signals, which are likely generated by the α-helical ππ* and nπ* transitions, respectively, overlapped with the β-sheet nπ* transition around 216 nm [52]. The recorded spectra were similar to previous far-UV CD measurements of RTA [17]–[20], [51] and, consistent with the crystal structure of ricin (PDB entry 1AAI; [53]), indicated an α/β-type structure. The RTA spectrum was slightly red shifted in the presence of PBA, which may indirectly indicate hydrophobic interactions between the protein and PBA resulting in a less polar microenvironment [52]. Thus, PBA treatment did not induce gross structural alterations to RTA. Thermal unfolding profiles derived from the raw data were used to determine the secondary structure transition temperature (*T*_m_; the midpoint between folded and unfolded conformations) for untreated and PBA-treated toxin (Fig. 2C). Untreated RTA exhibited a *T*_m_ of 44.2°C (Table 1). PBA shifted the temperature of RTA thermal unfolding to a higher temperature of 48.5°C and thus exerted a stabilizing effect on the protein (Table 1).

**Table 1:** Influence of PBA, POPC/POPG (4∶1), and Glycerol on the Thermal Stability of RTA a.

Condition	*T*_m_ (°C)
No treatment	44.2±0.7
+ PBA	48.5±0.5
+ POPC/POPG	27.4±1.0
+ POPC/POPG & PBA	33.4±0.5
+ Glycerol	49.3±0.8
+ Glycerol & POPC/POPG	43.5±1.0

a *T*_m_ values were calculated from the thermal unfolding profiles presented in Figures 2, 4, and 7. Values represent the averages ± ranges from two independent experiments per condition.

**Figure 3:**
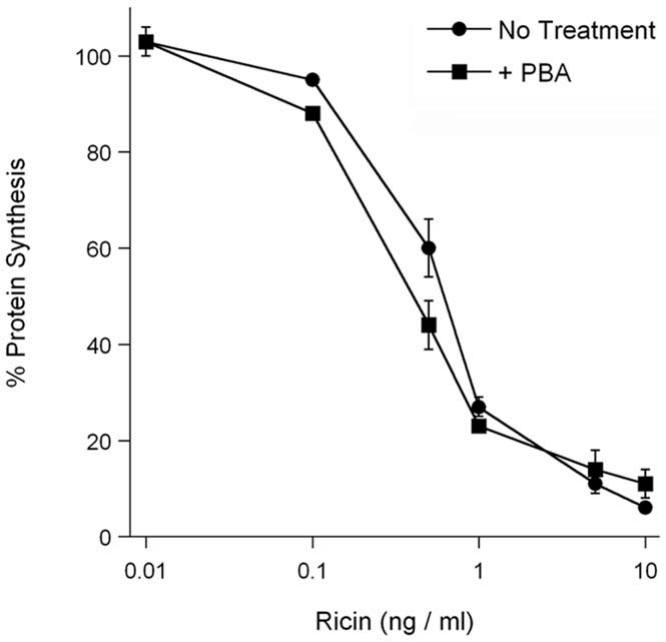
PBA does not inhibit ricin intoxication. Vero-d2EGFP cells were exposed to the indicated concentrations of ricin for 16 h in the absence (circles) or presence (squares) of 100 µM PBA. The means ± standard errors of the means of four independent experiments with six replicate samples for each condition are shown.

We have previously shown that PBA prevents the thermal unfolding of CTA1, which in turn blocks the ER-to-cytosol export of CTA1 and productive intoxication [47]. The unfolding of RTA also occurs before toxin translocation to the cytosol [2], [16], [19], [20], so we predicted that PBA would inhibit ricin intoxication as well. To test this prediction, Vero-d2EGFP cells were incubated with 100 µM PBA and various concentrations of ricin for 18 hours. The cytotoxic effect of ricin prevents d2EGFP synthesis, so the fluorescent signal from Vero-d2EGFP cells decays over time. Loss of fluorescence is thus used as an indicator of intoxication [49], [50]. Surprisingly, rather than protect cells, PBA treatment slightly sensitized cells to ricin challenge by an unknown mechanism (Fig. 3).

**Figure 4:**
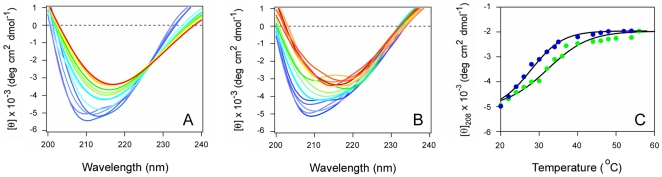
POPC/POPG destabilizes RTA in either the absence or presence of PBA. (A–B): The temperature-induced unfolding of RTA secondary structure in the presence of POPC/POPG (4∶1 molar ratio) vesicles (A) or in the presence of both POPC/POPG vesicles and PBA (B) was monitored by far-UV CD. In panel (B), LUVs were introduced 30 min after toxin exposure to 100 µM PBA at 20°C. Data were recorded with 2 µM RTA in pH 7.2 buffer. The change in color from blue to red corresponds to a change in temperature from 20°C to 60°C. (C): The mean residue molar ellipticities at 208 nm ([θ]_208_) in the presence of either POPC/POPG (blue) or both POPC/POPG and PBA (green) were plotted as a function of temperature.

RTA is destabilized by an interaction with negatively charged phospholipid membranes which have been used to mimic the inner leaflet of the ER membrane [19], [20]. We hypothesized that this interaction nullified the stabilizing effect of PBA on RTA and thus allowed productive intoxication of PBA-treated cells. To test this prediction, we monitored the effect of phospholipid vesicles containing the anionic lipid POPG on the thermal unfolding of RTA in the either the absence or presence of PBA (Fig. 4). In the presence of LUVs containing 20% POPG + 80% POPC, the thermal unfolding of RTA secondary structure exhibited a dramatically decreased *T*_m_ of 27.4°C (Fig. 4A, 4C, and Table 1). When exposed to both POPC/POPG vesicles and 100 µM PBA, the thermal transition for the secondary structure of RTA exhibited a *T*_m_ of 33.4°C (Fig. 4B–C, Table 1). These data indicated that anionic membranes exert a strong destabilizing effect on RTA secondary structure. Furthermore, PBA only partially restored the thermal stability of RTA: in the presence of both POPC/POPG vesicles and PBA, the *T*_m_ was still significantly lower than that of the untreated protein (see Table 1). Thus, the stabilizing effect of PBA on RTA structure was largely negated by the destabilizing effect of anionic membranes. The inability of PBA to stabilize RTA in the presence of anionic lipids provided a molecular explanation for the failure of PBA to protect cultured cells from challenge with ricin holotoxin, where the toxin is exposed to the inner face of the negatively charged ER membrane.

**Figure 5:**
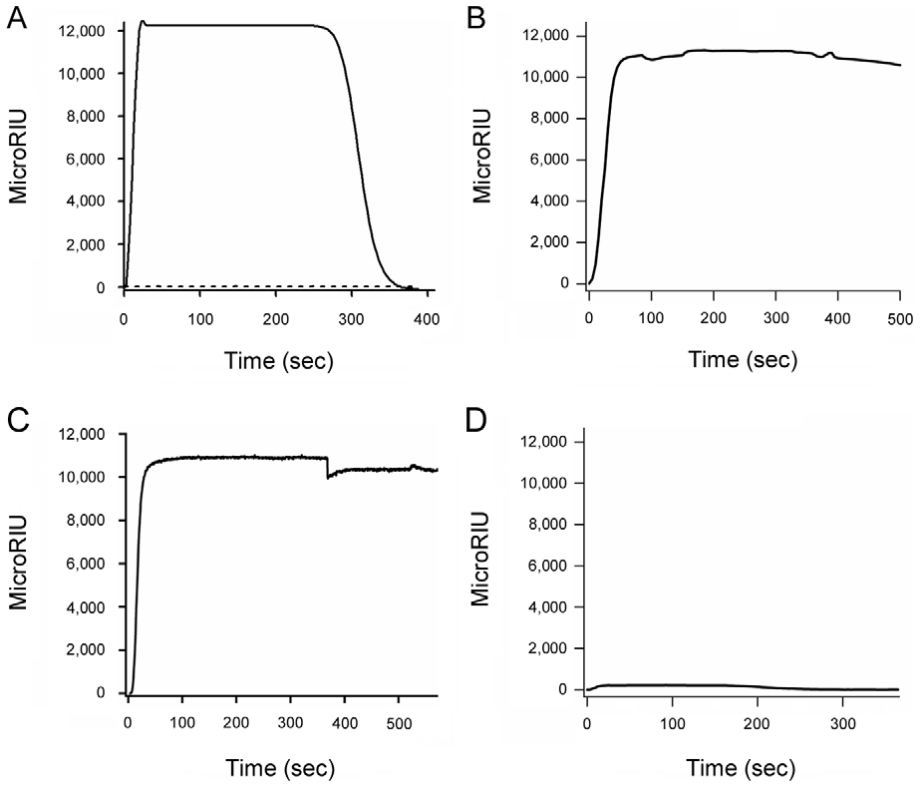
POPC/POPG treatment removes PBA from RTA. (A–C, solid lines): SPR sensor slides coated with RTA were exposed to perfusion buffer containing 100 µM PBA at 37°C for 300 sec. The buffer was then replaced with buffer containing (A) 100 µM PBA and 80% POPC : 20% POPG LUVs, (B) 100 µM PBA, or (C) 100 µM PBA and 100% POPC LUVs. As shown by the dotted line in panel A, control experiments demonstrated that LUVs alone did not generate a positive signal from the RTA sensor slide. (D): RTA was irreversibly denatured by a 1 hr, 50°C heat treatment and then appended to an SPR sensor slide. PBA was subsequently perfused over the slide at a concentration of 100 µM. For each slide, the baseline signal from the bound toxin was set at 0 microRIU before PBA perfusion.

The destabilizing effect of phospholipid vesicles on PBA-bound RTA might result from the phospholipid-mediated displacement of PBA from the toxin. SPR was used to examine this possibility (Fig. 5). PBA was perfused at 37°C over a sensor slide coated with RTA until the binding equilibrium was reached. Then, the perfusion buffer was replaced with a buffer containing both PBA and POPC/POPG vesicles. This led to rapid displacement of PBA from the sensor slide (Fig. 5A). POPC/POPG vesicles alone did not generate a signal when perfused over the RTA plate (Fig. 5A), possibly because anionic LUVs rupture upon contact with RTA [19] and would thus lack the necessary mass to alter the refractive index of the slide. The process of buffer switching alone was not responsible for displacement of RTA-bound PBA, as no substantial loss of signal occurred when the PBA-containing buffer was replaced with another PBA-containing buffer (Fig. 5B) or with a buffer containing both PBA and 100% POPC vesicles (Fig. 5C). The latter observation was consistent with the established lack of interaction between RTA and neutral phospholipid vesicles [19], [20]. Thus, POPC/POPG vesicles could effectively remove pre-bound PBA from RTA.

We further hypothesized that the POPC/POPG-induced destabilization of RTA was responsible for displacing pre-bound PBA. In this model, PBA would not bind to unfolded conformations of RTA. We tested this prediction by perfusing PBA over an SPR sensor coated with RTA that had been denatured by a one hour, 50°C heat treatment [16]. As shown in Figure 5D, PBA did not bind to denatured RTA. Our collective observations thus indicated that the destabilizing effect of anionic phospholipids is dominant over the stabilizing effect of PBA, and that the phospholipid-induced unfolding of RTA displaces pre-bound PBA.

**Figure 6:**
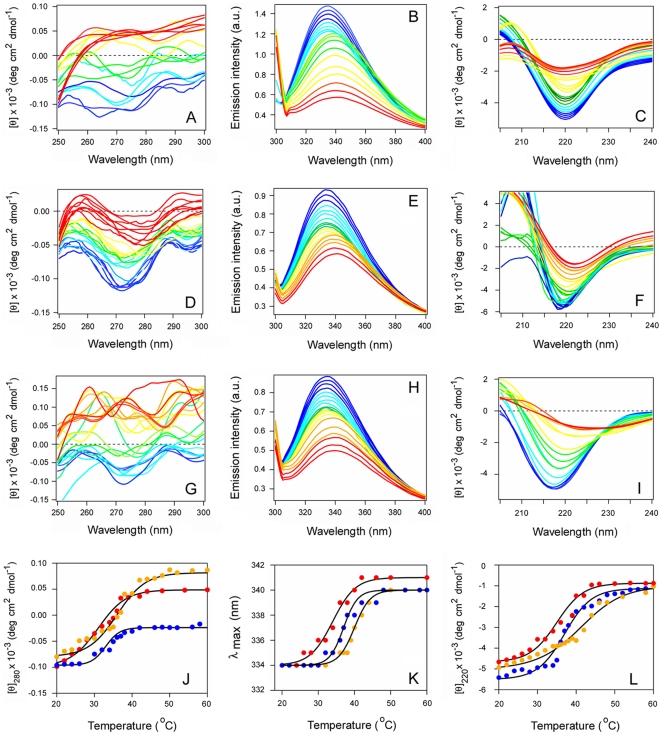
Anionic phospholipid vesicles do not destabilize CTA1 in either the absence or presence of PBA. (A–I): The temperature-induced unfolding of untreated CTA1 (A–C), CTA1 treated with POPC/POPG (at 4∶1 molar ratio) vesicles (D–F), or CTA1 treated with PBA and POPC/POPG vesicles (G–I) was monitored by near-UV CD (A, D, G), fluorescence spectroscopy (B, E, H), and far-UV CD (C, F, I). In panels (G–I), LUVs were introduced 30 min after toxin exposure to 100 µM PBA at 20°C. Data were recorded with 15 µM CTA1 in pH 7.2 buffer. The change in color from blue to red corresponds to a change in temperature from 20°C to 60°C. (J–L): Thermal unfolding profiles for CTA1 (red), CTA1 + lipid (blue), and CTA1 + PBA + lipid (yellow) were derived from the data presented in panels A–I. (J): For near-UV CD analysis, the mean residue molar ellipticities at 280 nm ([θ]_280_) were plotted as a function of temperature. (K): For fluorescence spectroscopy, the maximum emission wavelength (λ_max_) was plotted as a function of temperature. (L): For far-UV CD analysis, the mean residue molar ellipticities at 220 nm ([θ]_220_) were plotted as a function of temperature.

We have previously shown that PBA blocks the thermal unfolding of CTA1, the ER-to-cytosol export of CTA1, and CT intoxication [47]. These results suggested that, in contrast to RTA, anionic phospholipid membranes (such as the ER membrane) do not alter the impact of PBA on CTA1 stability. CD and fluorescence spectroscopy were used to test this prediction (Fig. 6). Consistent with previous reports [14], [35], [36], [47], the isolated CTA1 subunit was in a partially unfolded conformation at the physiological temperature of 37°C (Fig. 6A–C and J–L). CTA1 exhibited a tertiary structure *T*_m_ of 31.5°C, a *T*_m_ of 34.4°C for the red shift to the maximum emission wavelength (λ_max_) of tryptophan fluorescence, and a secondary structure *T*_m_ of 34.8°C (Table 2). POPC/POPG vesicles did not destabilize CTA1, but rather had a slight stabilizing effect (Fig. 6D–F): in the presence of these vesicles, the *T*_m_ values derived from far-UV CD, near-UV CD, and fluorescence experiments were shifted to 2–3°C higher temperatures than recorded for the control condition (Fig. 6J–L and Table 2). This stood in sharp contrast to the dramatic destabilizing effect of negatively charged vesicles on RTA thermal stability. When CTA1 was treated with both PBA and POPC/POPG vesicles (Fig. 6G–I), we recorded a 5–7°C increase in *T*_m_ values (Fig. 6 J–L and Table 2) that was similar to the stabilizing effect previously reported for PBA alone [47]. Collectively, these observations demonstrated that anionic phospholipid vesicles are neither a general protein destabilizer nor a direct inhibitor of PBA. The data also provided a molecular explanation for the differential effects of PBA on ricin vs. cholera intoxication: PBA does not inhibit ricin intoxication and does not prevent unfolding of RTA in the presence of anionic phospholipids, whereas PBA inhibits both CT intoxication and CTA1 unfolding in the presence of negatively charged phospholipids at physiological temperature.

**Table 2:** Influence of PBA and POPC/POPG (4∶1) on the Thermal Stability of CTA1^a^.

	*T*_m_ (°C)		
Condition	near-UV CD	λ_max_	far-UV CD
No treatment	31.5±0.5	34.4±0.6	34.8±0.7
+ POPC/POPG	33.6±1.0	36.8±0.8	37.7±1.0
+ POPC/POPG & PBA	36.3±1.0	39.7±0.7	42.5±0.8

a *T*_m_ values were calculated from the thermal unfolding profiles presented in Figure 6. Values represent the averages ± ranges from two independent experiments per condition.

**Figure 7:**
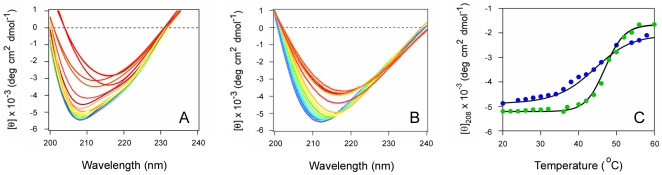
Glycerol prevents the thermal unfolding of RTA in either the absence or presence of POPC/POPG. (A–B): The temperature-induced unfolding of RTA secondary structure in the presence of 10% glycerol (A) or in the presence of both 10% glycerol and POPC/POPG (B) was monitored by far-UV CD. In panel (B), 100 nm LUVs containing 80% POPC and 20% POPG were introduced 30 min after toxin exposure to 10% glycerol at 20°C. Data were recorded with 2 µM RTA in pH 7.2 buffer. The change in color from blue to red corresponds to a change in temperature from 20°C to 60°C. (C): The mean residue molar ellipticities at 208 nm ([θ]_208_) in the presence of either 10% glycerol (green) or both 10% glycerol and POPC/POPG (blue) were plotted as a function of temperature.

Glycerol is a general protein stabilizer that confers cellular resistance to ricin, CT, and other AB toxins [35], [50], [54]. Glycerol has also been shown to prevent the thermal unfolding of CTA1 [35]. We accordingly predicted that glycerol would inhibit the thermal unfolding of RTA, and that negatively charged phospholipid vesicles would not block the stabilizing effect of glycerol. Far-UV CD was used to test this prediction (Fig. 7). Exposure to 10% glycerol resulted in a substantial increase in RTA thermal stability: the secondary structure *T*_m_ was increased to 49.3°C, which was 5.1°C higher than the *T*_m_ for untreated RTA (Fig. 7A, 7C, and Table 1). Consistent with our data, previous work reported that an incubation with 10% glycerol raises the *T*_m_ for the red shift to the λ_max_ of RTA tryptophan fluorescence by 4°C [18]. Exposure to both 10% glycerol and POPC/POPG vesicles (Fig. 7B) partially attenuated the stabilizing effect of glycerol: under this condition, RTA exhibited a secondary structure *T*_m_ of 43.5°C (Fig. 7C, Table 1). This *T*_m_ was similar to the *T*_m_ obtained from untreated RTA, but it was also 16.1°C higher than the *T*_m_ obtained for POPC/POPG-treated toxin (Table 1). Glycerol treatment, in contrast to PBA, thus prevented the destabilizing effect of anionic phospholipid vesicles on the structure of RTA. These observations provide a molecular explanation for the differential effects of PBA and glycerol on ricin intoxication: PBA did not inhibit ricin intoxication and did not affect toxin destabilization by anionic phospholipids, whereas glycerol inhibited both ricin intoxication and toxin destabilization by negatively charged phospholipids.

## Discussion

ER-translocating toxins bind to a variety of surface receptors, follow distinct trafficking routes to the ER, and modify their specific targets in the host cytosol. However, with the exception of cytolethal distending toxin [55], all known ER-translocating toxins appear to exploit the ERAD system for A chain translocation to the cytosol [2]. The inhibition of ERAD-mediated toxin translocation could thus confer broad-spectrum resistance to a subset of AB toxins.

It was originally thought that a hydrophobic domain near the C-terminus of the A chain allowed the toxin to masquerade as a misfolded protein for ERAD recognition and subsequent export to the cytosol [10]. More recent studies have suggested an alternative model in which the A chain actually assumes a disordered conformation upon its dissociation from the holotoxin at 37°C. Thermal instability in the isolated A chain has been reported for the catalytic subunits of PT, CT, and ricin [13], [14], [19], [20]. In contrast, the catalytic subunit of cytolethal distending toxin (which does not use ERAD to exit the ER) is thermally stable [56]. The inhibition of A chain unfolding at physiological temperature could thus block the ERAD-mediated translocation of multiple AB toxins.

We have previously shown that either 10% glycerol or 100 µM PBA will inhibit the thermal disordering of CTA1, the ER-to-cytosol export of CTA1, and CT intoxication [35], [47]. PBA is a chemical chaperone and an FDA-approved therapeutic for the treatment of urea cycle disorders [46], [57]. It therefore held promise as a drug that could generate broad-spectrum toxin resistance through an inhibition of A chain unfolding. The potential stabilizing effect of PBA on A chain structure could also help improve the expression and storage of recombinant RTA vaccines. Our data indicate that PBA substantially increases the thermal stability of RTA and, thus, could potentially be used in the formulation of RTA vaccines. A 4.3°C increase in the secondary structure *T*_m_ of RTA was obtained with 100 µM PBA (Fig. 2), and a 7.8°C increase in the secondary structure *T*_m_ was obtained with 1 mM PBA (Fig. S2). PBA is thus more effective than any of the previous compounds evaluated as RTA stabilizers, with the exception of 50% glycerol [18]. Future studies will be required to determine whether this level of stabilization can aid long-term storage of lyophilized or soluble RTA as well as other vaccine antigens. In terms of vaccine development, our current observations represent a preliminary step that could orient further research on small molecule stabilizers of vaccine antigens.

PBA did not protect cultured cells from ricin intoxication (Fig. 3). The different effects of PBA on CT intoxication vs. ricin intoxication apparently result from the distinct host-toxin interactions that occur in the ER for these two toxins. CTA1 and RTA both use thermal instability as a means to activate the ERAD translocation mechanism. However, as highlighted in this work, the translocation of each toxin involves distinct molecular events. CTA1 has a disordered tertiary structure and a partially disturbed secondary structure at the physiological temperature of 37°C [14], so further host-induced unfolding is apparently unnecessary for its ERAD-mediated translocation. Indeed, we found that exposure to anionic phospholipids did not lead to further destabilization of CTA1 (Fig. 6). The PBA-induced stabilization of CTA1 can thus occur in vivo as well as in vitro, thereby preventing toxin export to the cytosol and productive intoxication [47]. In contrast, RTA is more stable than CTA1 and uses an interaction with the negatively charged phospholipids of the ER membrane to induce further unfolding [19], [20]. The destabilization by anionic phospholipids is dominant over the PBA-induced stabilization of RTA (Fig. 4), so PBA is unlikely to inhibit the in vivo unfolding and translocation of RTA that is exposed to the negatively charged ER membrane. The different pathways utilized by CTA1 and RTA to attain a disordered, translocation-competent conformation thus produce different outcomes when PBA is applied in vivo to block intoxication.

Treatment with 10% glycerol stabilized RTA in both the absence and presence of anionic phospholipids (Fig. 7). This condition is known to inhibit ricin intoxication [54] (S. Massey and K. Teter, unpublished observations), so the general strategy of toxin stabilization appears to be a valid therapeutic approach. Furthermore, the glycerol-induced block of ricin intoxication strongly suggests that the unfolding of RTA by anionic phospholipids is a key step for toxin translocation. RTA exposed to both glycerol and POPC/POPG vesicles exhibited about the same secondary structure *T*_m_ as untreated RTA (Table 1), which indicates the intrinsic thermal instability of RTA is insufficient to promote toxin translocation and productive intoxication. The possible extent of host-assisted A chain unfolding was further documented by the dramatic ∼17°C decrease in secondary structure *T*_m_ for POPC/POPG-treated RTA. These collective observations provide further support for a previously suggested model in which A chain interaction with the ER membrane is an essential event for ricin translocation to the cytosol [20].

Preliminary experiments have shown that PBA also fails to protect cultured cells from ST (Fig. S3). In contrast, glycerol-treated cells are resistant to ST [50]. These observations mirror the results obtained with ricin and suggest that STA1 unfolding also involves an interaction with the negatively charged phospholipids of the ER membrane. Consistent with this model, it has been shown that (i) the C-terminus of STA1 binds to membranes containing 20–30% anionic phospholipids [24], [25] and (ii) the C-terminus of STA1 is required for productive intoxication [22], [25]. Likewise, the C-terminus of RTA appears to mediate the interaction with anionic phospholipids which results in its unfolding [20], [21]. Computational predictions of toxin stability further indicate that STA1 is more stable than CTA1 and is either as stable or more stable than RTA, depending on the STA1 variant (Table S1). Based on these observations, we hypothesize that, like RTA, physiological temperature alone is not sufficient to place the dissociated STA1 subunit in a disordered conformation for ERAD recognition.

Our collective data indicate that, similar to the numerous AB toxin trafficking routes from the cell surface to the ER, the ERAD-mediated translocation of toxin A chains from the ER to the cytosol is a heterogeneous process. For some toxins, A chain thermal instability alone is sufficient to generate a disordered conformation for ERAD recognition. In other cases, ERAD recognition requires further destabilization of the A chain via an interaction with the ER membrane. Stabilization of the A chain will prevent ERAD-mediated export for either of the aforementioned categories of toxin, but the stabilizing agent must be able to supersede the host-toxin interactions which place the A chain in a translocation-competent conformation. The identification of such a non-toxic agent represents a major challenge for the development of a broad-spectrum inhibitor that blocks ER-localized toxin unfolding. Our data indicate PBA could potentially serve as a therapeutic agent for certain toxins such as CT, as well as a preservative in bacterial or plant toxin vaccine production such as in the case of RTA.

## Supporting Information

Figure S1 **Intracellular transport of CT and ricin.** As reviewed in [29], [30], surface-bound toxins are internalized by receptor-mediated endocytosis. A substantial portion of internalized toxin is directed to the lysosomes for degradation (long, skinny arrow). The functional pool of internalized toxin moves through two early endosome compartments (sorting and recycling endosomes) en route to the *trans*-Golgi network. An additional vesicle trafficking step delivers the toxin to the ER in a process that may bypass the Golgi apparatus. The intact AB toxin cycles between the Golgi apparatus and ER until holotoxin disassembly in the ER releases the A subunit (red oval) from the membrane-associated B subunit (blue oval). The holotoxin-associated A chain is held in a stable conformation [33], [34], but the isolated A chain is an unstable protein that will unfold in the ER at 37°C [14], [20]. This unfolding event identifies the dissociated A chain as a substrate for ERAD-mediated translocation to the cytosol. An interaction with host factors in the cytosol allows the translocated A chain to regain a folded, active conformation [14]–[16]. The isolated B subunit can continue to cycle between the ER and Golgi apparatus; its ultimate fate remains unknown. Although CT and ricin pass through numerous organelles of varying pH, only conditions in the ER affect the unfolding of the A chain: alkalinization of the endomembrane system inhibits neither CT nor ricin toxicity [27], [28].
(TIF)

Figure S2 **Dose-dependent inhibition of RTA** **unfolding by PBA.** (A): Far-UV CD measurements of RTA secondary structure were taken in the presence of 1 mM PBA. Data were recorded with 2 µM RTA in pH 7.2 buffer. The change in color from blue to red corresponds to a change in temperature from 20°C to 60°C. (B): The mean residue molar ellipticities at 208 nm ([θ]_208_) were plotted as a function of temperature. RTA exposed to 1 mM PBA exhibited a secondary structure *T*_m_ of 52°C, which was 7.8°C higher than the *T*_m_ for untreated RTA and 3.5°C higher than the *T*_m_ for RTA treated with 100 µM PBA.
(TIF)

Figure S3 **PBA does not inhibit the cytotoxic activity of ST.** Vero-d2EGFP cells were exposed to 10-fold dilutions of an *E. coli* culture supernatant containing ST1 and ST2 for 16 h in the absence (circles) or presence (squares) of 100 µM PBA. The means ± standard errors of the means of four independent experiments with six replicate samples for each condition are shown.
(TIF)

Table S1 **Computational predictions of toxin stability.** Protein instability data for the A chains of Shiga toxin (STA1), Shiga-like toxin 1 (ST1 A1), Shiga-like toxin 2 (ST2 A1), ricin (RTA), cholera toxin (CTA1), *E. coli* heat-labile toxin (LTA1), and pertussis toxin (PT S1) were obtained from the ProtParam function of ExPASy-SWISS-PROT. An instability index value greater than 40 is indicative of protein instability.
(DOC)

## References

[1] Sandvig K, van Deurs B (2002). Membrane traffic exploited by protein toxins.. Annu Rev Cell Dev Biol.

[2] Lord JM, Roberts LM, Lencer WI (2005). Entry of protein toxins into mammalian cells by crossing the endoplasmic reticulum membrane: co-opting basic mechanisms of endoplasmic reticulum-associated degradation.. Curr Top Microbiol Immunol.

[3] Spooner RA, Watson PD, Marsden CJ, Smith DC, Moore KA (2004). Protein disulphide-isomerase reduces ricin to its A and B chains in the endoplasmic reticulum.. Biochem J.

[4] Hazes B, Boodhoo A, Cockle SA, Read RJ (1996). Crystal structure of the pertussis toxin-ATP complex: a molecular sensor.. J Mol Biol.

[5] Majoul I, Ferrari D, Soling HD (1997). Reduction of protein disulfide bonds in an oxidizing environment. The disulfide bridge of cholera toxin A-subunit is reduced in the endoplasmic reticulum.. FEBS Lett.

[6] Orlandi PA (1997). Protein-disulfide isomerase-mediated reduction of the A subunit of cholera toxin in a human intestinal cell line.. J Biol Chem.

[7] Tsai B, Rodighiero C, Lencer WI, Rapoport TA (2001). Protein disulfide isomerase acts as a redox-dependent chaperone to unfold cholera toxin.. Cell.

[8] Brodsky JL (2007). The protective and destructive roles played by molecular chaperones during ERAD (endoplasmic-reticulum-associated degradation).. Biochem J.

[9] Deeks ED, Cook JP, Day PJ, Smith DC, Roberts LM (2002). The low lysine content of ricin A chain reduces the risk of proteolytic degradation after translocation from the endoplasmic reticulum to the cytosol.. Biochemistry.

[10] Hazes B, Read RJ (1997). Accumulating evidence suggests that several AB-toxins subvert the endoplasmic reticulum-associated protein degradation pathway to enter target cells.. Biochemistry.

[11] Rodighiero C, Tsai B, Rapoport TA, Lencer WI (2002). Role of ubiquitination in retro-translocation of cholera toxin and escape of cytosolic degradation.. EMBO Rep.

[12] Worthington ZE, Carbonetti NH (2007). Evading the proteasome: absence of lysine residues contributes to pertussis toxin activity by evasion of proteasome degradation.. Infect Immun.

[13] Pande AH, Moe D, Jamnadas M, Tatulian SA, Teter K (2006). The pertussis toxin S1 subunit is a thermally unstable protein susceptible to degradation by the 20S proteasome.. Biochemistry.

[14] Pande AH, Scaglione P, Taylor M, Nemec KN, Tuthill S (2007). Conformational instability of the cholera toxin A1 polypeptide.. J Mol Biol.

[15] Ampapathi RS, Creath AL, Lou DI, Craft JW, Blanke SR (2008). Order-disorder-order transitions mediate the activation of cholera toxin.. J Mol Biol.

[16] Argent RH, Parrott AM, Day PJ, Roberts LM, Stockley PG (2000). Ribosome-mediated folding of partially unfolded ricin A-chain.. J Biol Chem.

[17] Olson MA, Carra JH, Roxas-Duncan V, Wannemacher RW, Smith LA (2004). Finding a new vaccine in the ricin protein fold.. Protein Eng Des Sel.

[18] Peek LJ, Brey RN, Middaugh CR (2007). A rapid, three-step process for the preformulation of a recombinant ricin toxin A-chain vaccine.. J Pharm Sci.

[19] Day PJ, Pinheiro TJ, Roberts LM, Lord JM (2002). Binding of ricin A-chain to negatively charged phospholipid vesicles leads to protein structural changes and destabilizes the lipid bilayer.. Biochemistry.

[20] Mayerhofer PU, Cook JP, Wahlman J, Pinheiro TT, Moore KA (2009). Ricin A chain insertion into endoplasmic reticulum membranes is triggered by a temperature increase to 37 {degrees}C.. J Biol Chem.

[21] Simpson JC, Lord JM, Roberts LM (1995). Point mutations in the hydrophobic C-terminal region of ricin A chain indicate that Pro250 plays a key role in membrane translocation.. Eur J Biochem.

[22] LaPointe P, Wei X, Gariepy J (2005). A role for the protease-sensitive loop region of Shiga-like toxin 1 in the retrotranslocation of its A1 domain from the endoplasmic reticulum lumen.. J Biol Chem.

[23] Menikh A, Saleh MT, Gariepy J, Boggs JM (1997). Orientation in lipid bilayers of a synthetic peptide representing the C-terminus of the A1 domain of shiga toxin. A polarized ATR-FTIR study.. Biochemistry.

[24] Saleh MT, Ferguson J, Boggs JM, Gariepy J (1996). Insertion and orientation of a synthetic peptide representing the C-terminus of the A1 domain of Shiga toxin into phospholipid membranes.. Biochemistry.

[25] Suhan ML, Hovde CJ (1998). Disruption of an internal membrane-spanning region in Shiga toxin 1 reduces cytotoxicity.. Infect Immun.

[26] Orlandi PA, Curran PK, Fishman PH (1993). Brefeldin A blocks the response of cultured cells to cholera toxin. Implications for intracellular trafficking in toxin action.. J Biol Chem.

[27] Lencer WI, Strohmeier G, Moe S, Carlson SL, Constable CT (1995). Signal transduction by cholera toxin: processing in vesicular compartments does not require acidification.. Am J Physiol.

[28] Yoshida T, Chen CH, Zhang MS, Wu HC (1990). Increased cytotoxicity of ricin in a putative Golgi-defective mutant of Chinese hamster ovary cell.. Exp Cell Res.

[29] Lord JM, Spooner RA (2011). Ricin trafficking in plant and mammalian cells.. Toxins.

[30] Wernick NLB, Chinnapen DJ-F, Cho JA, Lencer WI (2010). Cholera toxin: an intracellular journey into the cytosol by way of the endoplasmic reticulum.. Toxins.

[31] Wu MM, Llopis J, Adams SR, McCaffery JM, Teter K (2000). Studying organelle physiology with fusion protein-targeted avidin and fluorescent biotin conjugates.. Methods Enzymol.

[32] Kim JH, Johannes L, Goud B, Antony C, Lingwood CA (1998). Noninvasive measurement of the pH of the endoplasmic reticulum at rest and during calcium release.. Proc Natl Acad Sci U S A.

[33] Goins B, Freire E (1988). Thermal stability and intersubunit interactions of cholera toxin in solution and in association with its cell-surface receptor ganglioside GM1.. Biochemistry.

[34] Jackson LS, Tolleson WH, Chirtel SJ (2006). Thermal Inactivation of Ricin Using Infant Formula as a Food Matrix.. J Agric Food Chem.

[35] Massey S, Banerjee T, Pande AH, Taylor M, Tatulian SA (2009). Stabilization of the tertiary structure of the cholera toxin A1 subunit inhibits toxin dislocation and cellular intoxication.. J Mol Biol.

[36] Banerjee T, Pande A, Jobling MG, Taylor M, Massey S (2010). Contribution of subdomain structure to the thermal stability of the cholera toxin A1 subunit.. Biochemistry.

[37] Presley JF, Mayor S, Dunn KW, Johnson LS, McGraw TE (1993). The End2 mutation in CHO cells slows the exit of transferrin receptors from the recycling compartment but bulk membrane recycling is unaffected.. J Cell Biol.

[38] Audi J, Belson M, Patel M, Schier J, Osterloh J (2005). Ricin poisoning: a comprehensive review.. JAMA.

[39] O'Hare M, Roberts LM, Thorpe PE, Watson GJ, Prior B (1987). Expression of ricin A chain in *Escherichia coli*.. FEBS Lett.

[40] Piatak M, Lane JA, Laird W, Bjorn MJ, Wang A (1988). Expression of soluble and fully functional ricin A chain in *Escherichia coli* is temperature-sensitive.. J Biol Chem.

[41] Brandau DT, Jones LS, Wiethoff CM, Rexroad J, Middaugh CR (2003). Thermal stability of vaccines.. J Pharm Sci.

[42] Compton JR, Legler PM, Clingan BV, Olson MA, Millard CB (2010). Introduction of a disulfide bond leads to stabilization and crystallization of a ricin immunogen.. Proteins.

[43] Smallshaw JE, Richardson JA, Pincus S, Schindler J, Vitetta ES (2005). Preclinical toxicity and efficacy testing of RiVax, a recombinant protein vaccine against ricin.. Vaccine.

[44] Vitetta ES, Smallshaw JE, Coleman E, Jafri H, Foster C (2006). A pilot clinical trial of a recombinant ricin vaccine in normal humans.. Proc Natl Acad Sci U S A.

[45] Smallshaw JE, Vitetta ES (2010). A lyophilized formulation of RiVax, a recombinant ricin subunit vaccine, retains immunogenicity.. Vaccine.

[46] Maestri NE, Brusilow SW, Clissold DB, Bassett SS (1996). Long-term treatment of girls with ornithine transcarbamylase deficiency.. N Engl J Med.

[47] Taylor M, Banerjee T, Navarro-Garcia F, Huerta J, Massey S (2011). A therapeutic chemical chaperone inhibits cholera intoxication and unfolding/translocation of the cholera toxin A1 subunit.. PLoS ONE.

[48] Myszka DG (1997). Kinetic analysis of macromolecular interactions using surface plasmon resonance biosensors.. Curr Opin Biotechnol.

[49] Massey S, Quinones B, Teter K (2011). A Cell-Based Fluorescent Assay to Detect the Activity of Shiga Toxin and Other Toxins that Inhibit Protein Synthesis.. Methods Mol Biol.

[50] Quinones B, Massey S, Friedman M, Swimley MS, Teter K (2009). Novel cell-based method to detect Shiga toxin 2 from *Escherichia coli* O157:H7 and inhibitors of toxin activity.. Appl Environ Microbiol.

[51] Wawrzynczak EJ, Drake AF, Thorpe PE (1988). Circular dichroism of isolated ricin A- and B-chains.. Biophys Chem.

[52] Sreerama N, Woody RW, Berova N, Nakanishi,  K, Woody RW (2000). Circular dichroism of peptides and proteins., in *Circular Dichroism: Principles and Applications*.

[53] Rutenber E, Katzin BJ, Ernst S, Collins EJ, Mlsna D (1991). Crystallographic refinement of ricin to 2.5 A.. Proteins.

[54] Sandvig K, Madshus IH, Olsnes S (1984). Dimethyl sulphoxide protects cells against polypeptide toxins and poliovirus.. Biochem J.

[55] Guerra L, Teter K, Lilley BN, Stenerlow B, Holmes RK (2005). Cellular internalization of cytolethal distending toxin: a new end to a known pathway.. Cell Microbiol.

[56] Guerra L, Nemec KN, Massey S, Tatulian SA, Thelestam M (2009). A novel mode of translocation for cytolethal distending toxin.. Biochim Biophys Acta.

[57] Perlmutter DH (2002). Chemical chaperones: a pharmacological strategy for disorders of protein folding and trafficking.. Pediatr Res.

