# Supplementary figures and images for: Modulation of Toxin Stability by 4-Phenylbutyric Acid and Negatively Charged Phospholipids
Source: PLoS One. 2011 Aug 22;6(8):e23692. doi: 10.1371/journal.pone.0023692 (PMC3161752; doi:10.1371/journal.pone.0023692)

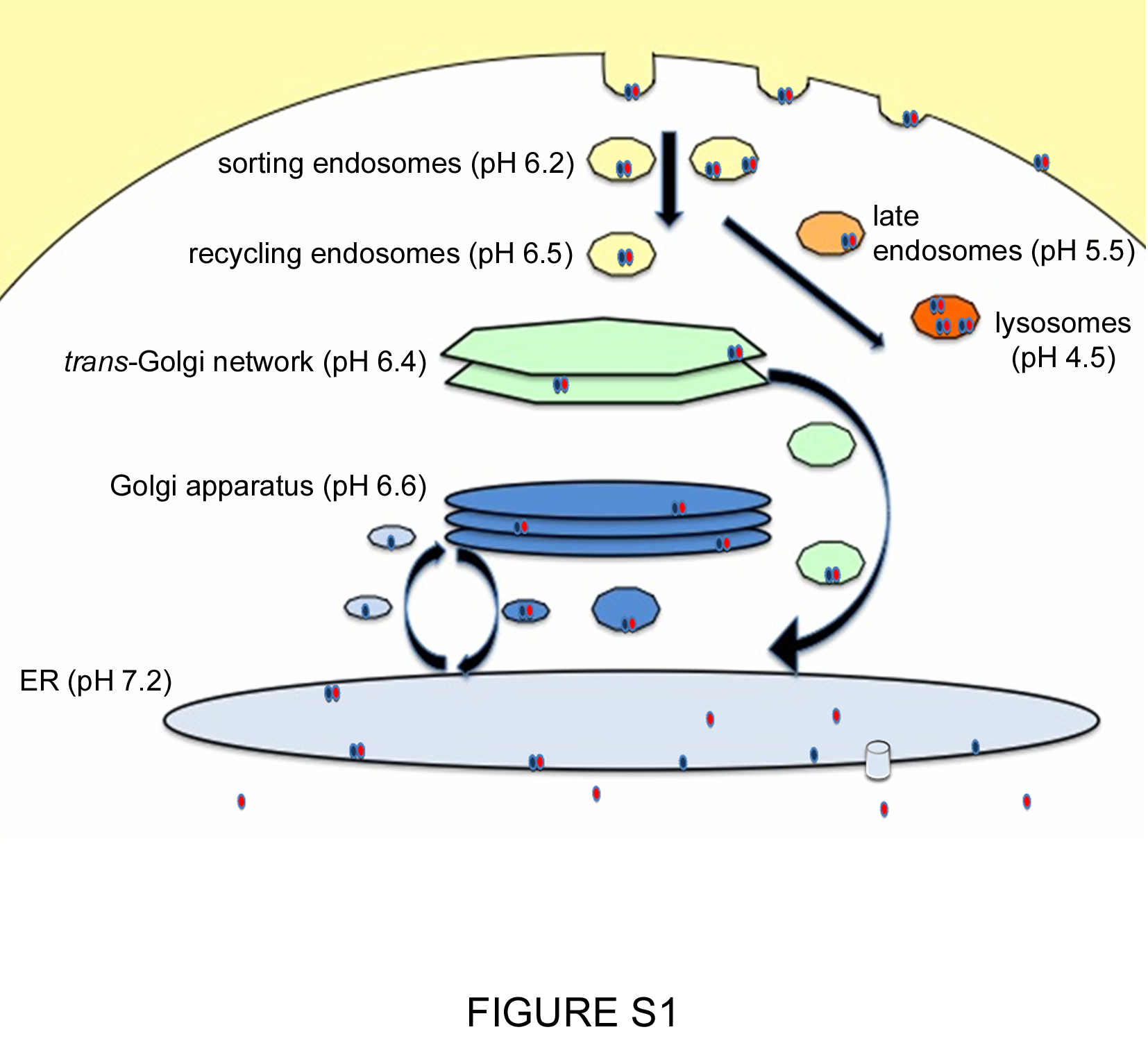

Supplement: Figure S1 — Intracellular transport of CT and ricin. As reviewed in [29], [30], surface-bound toxins are internalized by receptor-mediated endocytosis. A substantial portion of internalized toxin is directed to the lysosomes for degradation (long, skinny arrow). The functional pool of internalized toxin moves through two early endosome compartments (sorting and recycling endosomes) en route to the trans-Golgi network. An additional vesicle trafficking step delivers the toxin to the ER in a process that may bypass the Golgi apparatus. The intact AB toxin cycles between the Golgi apparatus and ER until holotoxin disassembly in the ER releases the A subunit (red oval) from the membrane-associated B subunit (blue oval). The holotoxin-associated A chain is held in a stable conformation [33], [34], but the isolated A chain is an unstable protein that will unfold in the ER at 37°C [14], [20]. This unfolding event identifies the dissociated A chain as a substrate for ERAD-mediated translocation to the cytosol. An interaction with host factors in the cytosol allows the translocated A chain to regain a folded, active conformation [14]–[16]. The isolated B subunit can continue to cycle between the ER and Golgi apparatus; its ultimate fate remains unknown. Although CT and ricin pass through numerous organelles of varying pH, only conditions in the ER affect the unfolding of the A chain: alkalinization of the endomembrane system inhibits neither CT nor ricin toxicity [27], [28]. (TIF) [file pone.0023692.s001.tif]

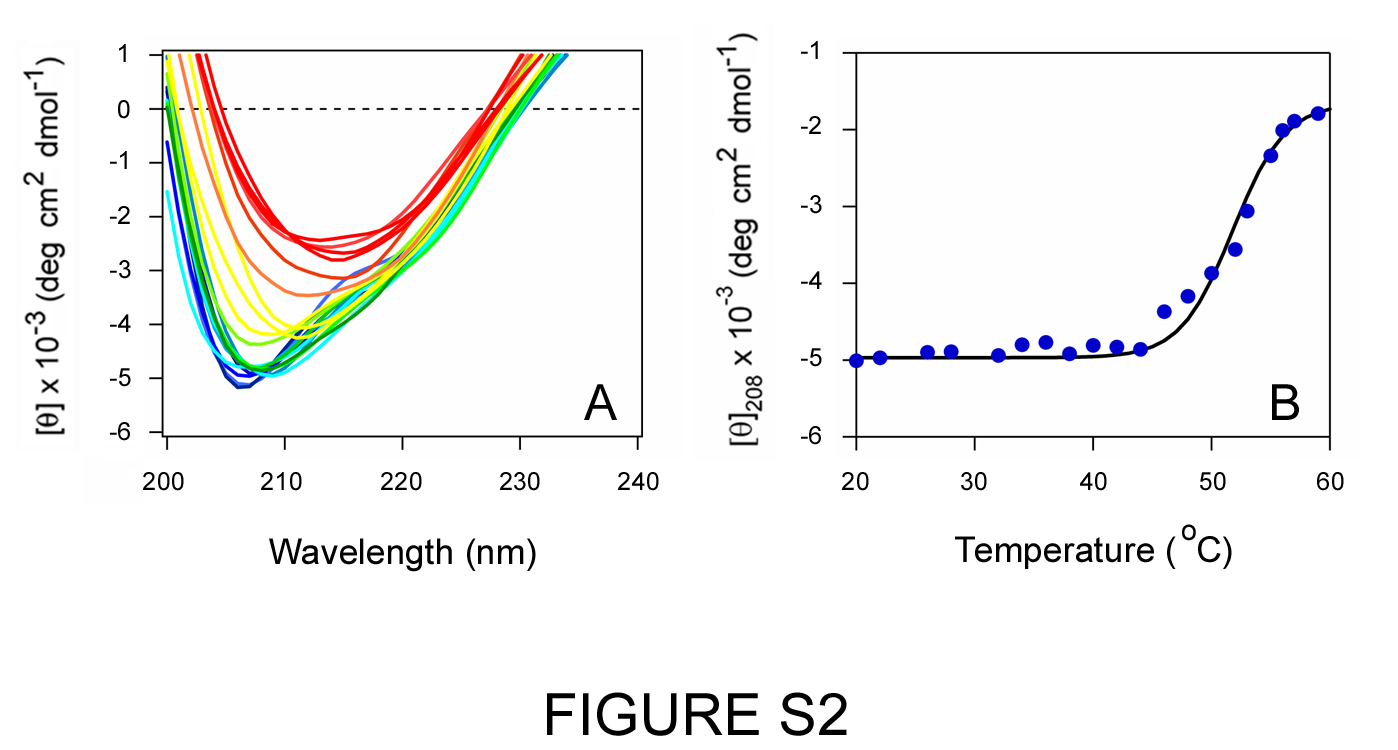

Supplement: Figure S2 — Dose-dependent inhibition of RTA unfolding by PBA. (A): Far-UV CD measurements of RTA secondary structure were taken in the presence of 1 mM PBA. Data were recorded with 2 µM RTA in pH 7.2 buffer. The change in color from blue to red corresponds to a change in temperature from 20°C to 60°C. (B): The mean residue molar ellipticities at 208 nm ([θ]208) were plotted as a function of temperature. RTA exposed to 1 mM PBA exhibited a secondary structure T m of 52°C, which was 7.8°C higher than the T m for untreated RTA and 3.5°C higher than the T m for RTA treated with 100 µM PBA. (TIF) [file pone.0023692.s002.tif]

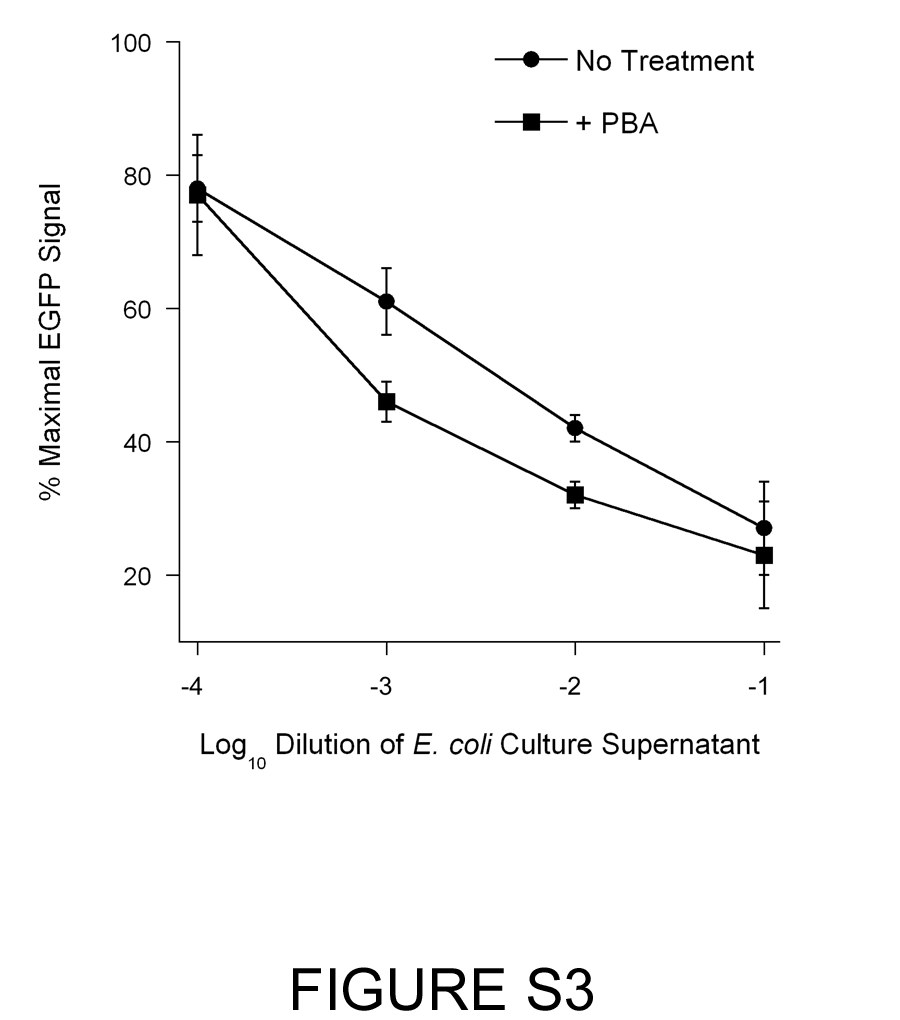

Supplement: Figure S3 — PBA does not inhibit the cytotoxic activity of ST. Vero-d2EGFP cells were exposed to 10-fold dilutions of an E. coli culture supernatant containing ST1 and ST2 for 16 h in the absence (circles) or presence (squares) of 100 µM PBA. The means ± standard errors of the means of four independent experiments with six replicate samples for each condition are shown. (TIF) [file pone.0023692.s003.tif]
