# Supplementary material for: Modulation of Toxin Stability by 4-Phenylbutyric Acid and Negatively Charged Phospholipids
Source: PLoS One. 2011 Aug 22;6(8):e23692. doi: 10.1371/journal.pone.0023692 (PMC3161752; doi:10.1371/journal.pone.0023692)
Supplement: Table S1 — Computational predictions of toxin stability. Protein instability data for the A chains of Shiga toxin (STA1), Shiga-like toxin 1 (ST1 A1), Shiga-like toxin 2 (ST2 A1), ricin (RTA), cholera toxin (CTA1), E. coli heat-labile toxin (LTA1), and pertussis toxin (PT S1) were obtained from the ProtParam function of ExPASy-SWISS-PROT. An instability index value greater than 40 is indicative of protein instability. (DOC) [file pone.0023692.s004.doc]

| Toxin | Instability Index | Classification |
| --- | --- | --- |
| STA1 | 28.3 | Stable |
| ST1 A1 | 29.0 | Stable |
| ST2 A1 | 35.6 | Stable |
| RTA | 35.5 | Stable |
| CTA1 | 41.6 | Unstable |
| LTA1 | 42.2 | Unstable |
| PT S1 | 45.2 | Unstable |
